# Advanced Glycation End-Products in Skeletal Muscle Aging

**DOI:** 10.3390/bioengineering8110168

**Published:** 2021-11-01

**Authors:** Lucas C. Olson, James T. Redden, Zvi Schwartz, David J. Cohen, Michael J. McClure

**Affiliations:** 1 Department of Biomedical Engineering, College of Engineering, Virginia Commonwealth University, Richmond, VA 23284, USA; olsonlc@vcu.edu (L.C.O.); reddenjt@vcu.edu (J.T.R.); zschwartz@vcu.edu (Z.S.); djcohen@vcu.edu (D.J.C.); 2 Department of Gerontology, College of Health Professions, Virginia Commonwealth University, Richmond, VA 23298, USA; 3 Department of Periodontics, University of Texas Health Science Center at San Antonio, San Antonio, TX 78229, USA

**Keywords:** skeletal muscle aging, sarcopenia, advanced glycation end-products, collagen, collagen cross-linking, motor nerve

## Abstract

Advanced age causes skeletal muscle to undergo deleterious changes including muscle atrophy, fast-to-slow muscle fiber transition, and an increase in collagenous material that culminates in the age-dependent muscle wasting disease known as sarcopenia. Advanced glycation end-products (AGEs) non-enzymatically accumulate on the muscular collagens in old age via the Maillard reaction, potentiating the accumulation of intramuscular collagen and stiffening the microenvironment through collagen cross-linking. This review contextualizes known aspects of skeletal muscle extracellular matrix (ECM) aging, especially the role of collagens and AGE cross-linking, and underpins the motor nerve’s role in this aging process. Specific directions for future research are also discussed, with the understudied role of AGEs in skeletal muscle aging highlighted. Despite more than a half century of research, the role that intramuscular collagen aggregation and cross-linking plays in sarcopenia is well accepted yet not well integrated with current knowledge of AGE’s effects on muscle physiology. Furthermore, the possible impact that motor nerve aging has on intramuscular cross-linking and muscular AGE levels is posited.

## 1. Introduction

Musculoskeletal injury increases due to falls and other accidental injuries in old age, and age-dependent alterations to skeletal muscle structure and function are the primary causal factors of such incidents [1]. The etiology of muscle atrophy due to aging, or sarcopenia, includes loss in muscle strength, muscle fiber wasting, increased intramuscular connective tissue, and a disruption of the muscle stem cell population that results in muscle that is weaker, stiffer, and less able to regenerate [2,3,4,5,6,7,8]. Muscular aging is multifactorial, involving extrinsic and intrinsic mechanisms that attack both the cellular components and extracellular matrix (ECM). Advanced glycation end-products (AGEs), the final derivative of the Maillard or browning reaction, are known to accumulate in musculoskeletal tissues in old age and are thought to play a role in the development of sarcopenia [9,10,11,12]. AGEs preferentially accrue on the long-lived extracellular matrix (ECM) proteins, especially collagens, since their formation relies on its precursors’ stochastic reaction (glucose and proteins) via the Maillard reaction. In addition to having a long half-life, collagens are rich in repeating arginine and lysine amino acids that potentiate the reaction between collagen and AGE precursors, further predisposing collagen to these glycation cross-links [12]. Non-enzymatic cross-linking by AGEs decrease collagen’s susceptibility to degradation by matrix metalloproteinases, causing the build-up of collagen and subsequent stiffening of the usually pliant skeletal muscle ECM [13]. There are only a handful of reviews that focus on the aging and cross-linking of the sclerotic collagens in skeletal muscle, and even fewer that address the role of AGEs in sarcopenic decline [14,15,16,17,18,19]. Furthermore, it has been 10 years since the last time this topic was reviewed in-depth, thus a purview of the literature in this space is warranted [20]. In addition, muscle-nerve interactions play an integral role in muscle health, and a discussion of motor neurons is included in this review in support of the critical role that AGEs have in the aging motor endplate.

## 2. Skeletal Muscle and the Aging Extracellular Matrix

Skeletal muscle ECM is highly structured and regulated to maintain its force-bearing properties and spatial-temporal regulation of muscle-specific stem cells, muscle fibers, and a host of other cell types including fibroblasts, endothelial cells, and nerve fibers [13,21,22,23,24]. The entire muscle is encased by the epimysium, which consists of type I and III collagen fibers that run perpendicular to the muscle and are responsible for maintaining the muscle girth [13,25,26]. Skeletal muscle is further cordoned off by the perimysium into muscle fiber groupings known as fascicles. The perimysium consists of type I and III collagen that runs both parallel to the muscle fibers, coalescing at the tendons, and perpendicular collagen that resists fascicular expansion [12,25]. Each muscle fiber is surrounded by the endomysium, which includes the basal and reticular laminae, that contain collagen orientated perpendicular to the muscle fibers during contraction and longitudinally orientated during relaxation [27,28,29,30].

### 2.1. ECM Composition Is Unique to the Muscle Fiber Type

Oxidative muscle fibers (types 1 and 2a) have a rich vascular supply, and as such have higher amounts of collagen in their endomysium, especially type IV, compared to glycolytic fibers (type 2b) [31]. In addition, the basal lamina is made up of laminin, fibronectin, vitronectin, entactin/nidogen, collagen type VI, and proteoglycans and is a reservoir for many soluble factors (including transforming growth factor beta (TGF-β)) [32,33]. Between the basal lamina and the muscle fiber resides the satellite cell, or the adult resident muscle stem cell, and the satellite cell modifies and maintains its basal lamina niche through the excretion of MMPs and laminin [24,34,35,36]. The reticular lamina is rich in type 1 collagen and is in-between adjacent basal laminae and is responsible for force transfer from the fiber [13,32,37].

### 2.2. Satellite Cells and Aging

Adult myogenesis is a series of highly coordinated events that follow a defined transcriptional template [38]. Adult muscle stem cells are known as satellite cells, and they lie quiescent between the muscle fiber and its apposed basal lamina and highly express Pax7, a pioneering transcription factor that maintains the myogenic potential of the cells [34,38]. Upon activation due to injury or other disruptive event, the satellite cells undergoes asymmetrical apical-basal division where the daughter cell close to the muscle fiber begins to express MyoD and Myf5 as the cell closer to the basal lamina remains solely a Pax7 positive cell for maintenance of the satellite cell pool (self-renewal) [38,39,40]. Other satellite cells undergo symmetrical division where both daughter cells either remain solely Pax7 positive to renew the quiescent population or both become activated MyoD positive myoblasts. Due in part to Myf5 upregulation, the activated myoblasts proliferate to expand the available pool of regenerative cells. Over time, the proliferating myoblasts sequentially lose Pax7, Myf5, and MyoD expression as Myogenin (MyoG) begins to be upregulated. As a result, the committed myoblast phenotype emerges where the myoblasts are less proliferative and are preparing for fusion. MyoG supports the expression of Myosin Heavy Chain type 1 (MyHC1) and the other contractile proteins as the cells begin to fuse into muscle fibers [41,42,43]. Dysregulation of the myogenic program is known to occur in the aging process, resulting in homeostatic decompensation, whereby the expression of myogenic factors such as MyoD and MyoG are upregulated [44,45,46]. It is thought that homeostatic decompensation, in part, explains the aberrant activation and depletion of the satellite cell niche. However, the mechanisms behind age-dependent satellite cell decline are still being elucidated.

### 2.3. The AGE-RAGE Axis in Skeletal Muscle Aging

**Figure 1:**
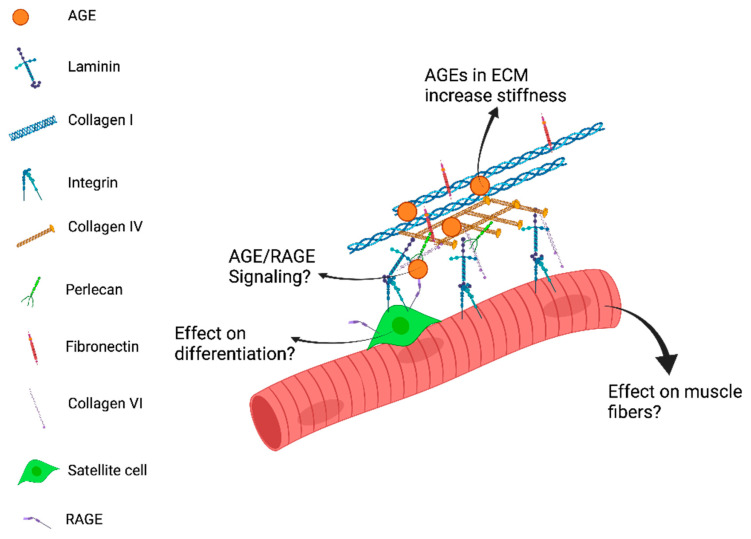
Advanced glycation end-products (AGEs) in skeletal muscle are associated with receptor for advanced glycation end-products (RAGE) activation and have been associated with muscle aging. RAGE plays a normal role in supporting adult myogenesis; however, when there is a pathological increase in RAGE activation due to AGEs, muscle wasting, increased muscular inflammation, and deleterious satellite cell signaling occurs. What remains unclear is whether effects from AGEs are due to soluble factors that activate RAGE signaling or from extracellular matrix cross-links that signal via RAGE as well. This figure proposes answered questions due to AGE cross-linking in muscle ECM.

The receptor for advanced-glycation end-products (RAGE) is a pattern-recognition receptor expressed in activated satellite cells and myoblasts, and was originally discovered in bovine lung homogenate due to its ability to bind AGEs [47,48]. RAGE is expressed in several cell types at a basal level [49,50,51]. However, it is not normally expressed in adult muscle fibers, but is upregulated after acute injury, in dystrophic muscles, and in cancer cachexia condition [48,52,53,54]. Further, RAGE signaling plays a normal role in skeletal muscle maintenance and regeneration [48]. Over time, other ligands in addition to AGEs were discovered such as amphoterin (HMGB1) and S100B, and it was observed that RAGE elicits a ligand-dependent response in cultured myoblasts [48,52,55,56,57,58]. Activated myoblasts respond to S100B via RAGE in the early phases of differentiation with enhanced proliferation via extracellular signal-regulated kinase 1/2 (ERK1/2) while priming the myogenic program through p38 mitogen activated protein kinase (MAPK) [58]. In the later phases of differentiation, HMGB1 upregulates myogenin via RAGE-dependent stimulation of p38 MAPK pathways, prepping the myoblasts for fusion by suppressing Pax7 [52]. In cases where AGEs are pathologically upregulated, such as in aging, AGE-RAGE signaling dominates normal RAGE processes, leading to an aberrant increase in satellite cell proliferation at the expense of differentiation (Figure 1) [48]. Moreover, systemic levels of soluble RAGE increase in individuals with sarcopenia [59]. AGE-RAGE signaling stimulates the phosphorylation of nuclear factor kappa B (NF-κB), upregulating extracellular matrix genes (e.g., collagen type I) and inflammatory cytokine production [60]. P-38 MAPK phosphorylation is upregulated by AGE-RAGE signaling and elevated p-38 MAPK signaling been implicated as a factor that disrupts satellite cell signaling in old age [52,58,61].

### 2.4. Aging of Skeletal Muscle Collagen

Collagen is the most abundant structural protein found in mammals, and is highly conserved between species [62,63,64]. As such, collagen aging in skeletal muscle has been investigated in humans, rats, mice, rabbits, lizards, seals, pigs, and cows [65,66,67,68,69,70,71,72,73]. A common feature is that the total amount of intramuscular collagen, as assayed by hydroxyproline, increases in old age [66,70]. In contrast, the acid-soluble fraction of collagen decreases in old age, indicating elevated collagen cross-linking [66,70,74]. As muscle develops, collagen is laid down and then fully cross-linked by the enzyme lysyl oxidase (LOX) which ceases by the end of sexual maturity [75]. Since the mature LOX cross-links (hydroxypyridine and pyridine) are enzymatic-dependent, they are destroyed and remade in humans following the normal 2 year half-life of skeletal muscle collagen (as determined by the ^14^C bomb-pulse technique) [75,76]. Interestingly, the half-life of skeletal muscle collagen increases with age, due to a reduction in the proteostasis of collagen [77]. Altogether, these phenomena indicate either a dysregulation in LOX-gelatinase control in the muscle or an increase in non-enzymatic cross-linking such as advanced glycation end-product (AGE) cross-linking. Gelatinases A and B (MMP-2 and MMP-9) lose their responsiveness to exercise-initiated ECM remodeling in old age [78]. Further, LOX protein levels increase in aged dystrophic mice, and there is a recent conference abstract reporting that LOX was increased in old female mice [79,80]. In addition, these data were correlated with decreased collagen solubility. However, there is opposing evidence found in a study on human muscle that the LOX-dependent cross-link hydroxylysylpyridinoline is unchanged in old age, while the AGE pentosidine is increased by 200% [65]. Furthermore, glycation cross-links were not investigated in the studies that measured LOX levels.

AGEs are non-enzymatic post-translational modifications to proteins in the body. AGE formation follows the stochastic browning, or Maillard, reaction where a monosaccharide (e.g., glucose, fructose, lactose, or ribose) in its open-ring conformation interacts with a protein to form an unstable Amadori product [9]. Over the course of a few days, the Amadori product matures and forms a very stable, irreversible AGE. Since AGE formation is a drawn-out, random process dependent on the concentration and probability of the monosaccharide existing in an open-ring conformation, most proteins in the body are degraded and turned over before appreciable AGE accumulation can occur. Due to collagen’s long life-span, AGEs are able to accumulate and have biological consequence resulting in stiffer collagen that is resistant to enzymatic degradation [12]. Furthermore, collagen is rich in lysine and arginine amino acids that potentiate AGE formation [12]. Interestingly, despite the high relevance of AGE’s role in skeletal muscle aging, there have been only a few studies characterizing the role of AGEs in this context [81,82].

### 2.5. Basal Lamina Aging

Age-dependent changes to the basal laminae alter muscle health, impact force production, and change how muscle stem cells respond to injury [20]. Many age-dependent changes in the composition and organization of the basal lamina occur. Fibronectin is depleted from the basal lamina in old age, reducing the integrin attachment points for satellite cells [22,83,84]. Laminin increases in fast-twitch muscle with age, while it is depleted from slow-twitch [85]. Additionally, collagen type VI, which acts as a bridge between the basal lamina and reticular lamina, is aberrantly increased in old age, disrupting the overall structure of the laminae [85]. Collagen type I increases significantly in the reticular laminae, and invades the basal laminar space. Furthermore, there is an increase in aberrant glycation cross-links that stiffens all collagens and other ECM components, which dysregulates quiescent muscle stem cells and muscle force transmission [65,72,86]. Collagen type IV, which forms a net-like structure in the basal lamina, serves as a scaffolding protein for the laminins and other cell-anchoring proteins [87]. Old age increases collagen type IV levels while decreasing the ability of muscle to regulate collagen type IV following injury [85,88]. AGE-modifications to ECM components reduces cell adhesion and disrupts the assembly of fibronectin to collagen type IV [89,90]. Further, AGE-modified basal lamina proteins caused mesangial cells to overexpress fibronectin matrix assembly, which implies that AGEs dysregulate the basal lamina structure [91].

### 2.6. ECM Aging Is Muscle-Specific

As mentioned previously, muscle ECM composition is highly dependent on the muscle fiber type. As such, muscles that are predominantly fast-twitch will age differently than muscles that are primarily slow-twitch. Thus, aging is dependent on the location of the muscle in the body, with the hind-limb muscles aging at a faster rate than the forelimb [7]. Fast-twitch muscles are especially sensitive to the aging process as there is a fast-to-slow muscle fiber transition that is coordinated by the aging of the motor neurons [8,12,56,92,93,94,95,96,97,98]. An immediate consequence of this is that these fast-twitch muscle become enriched with ECM components more typical of slow-twitch muscles, namely, the collagens [93,99]. Curiously, the fast-to-slow transition does not replicate the increased density of satellite cells found in slow-twitch muscles [21,100,101,102,103,104,105,106,107,108]. Increases in deleterious modifications to the ECM, such as cross-linking and an imbalance in ECM components, may be more responsible for the decrease in satellite cells seen in the fast-to-slow transition than the actual modification to the muscle fibers themselves. Slow-twitch muscles are more resistant to aging, and this is thought to be due to their role as postural muscles, which are constantly activated throughout life [109]. However, any fast-twitch glycolytic fibers found in slow-twitch muscles are reduced in old age [95]. AGE cross-linking accumulation is ubiquitous to slow and fast-twitch muscles, albeit there was a greater staining intensity observed in the myofibers of slow-twitch muscles [110]. However, no one has examined if the increased AGE presence in slow-twitch muscles is related to the increased collagen content, moreover, if the fast-to-slow twitch increase in collagen is partially driven by AGE cross-linking.

## 3. Peripheral Nerve Involvement in Aging and Atrophy

### 3.1. Overview and Neuromuscular Junction Anatomy

The neuromuscular junction (NMJ) is a highly regulated microenvironment maintained by cross-talk between glial, motor nerve, and skeletal muscle. The NMJ is comprised of three major segments, the pre-synaptic, post-synaptic, and synaptic extracellular space. Vesicle packaged acetylcholine is localized in the pre-synaptic space inside the peripheral nerve terminal. Following membrane depolarization, acetylcholine is released into the synaptic cleft binding to the acetylcholine receptor, activating sodium ion channels, which results in skeletal muscle contraction.

**Figure 2:**
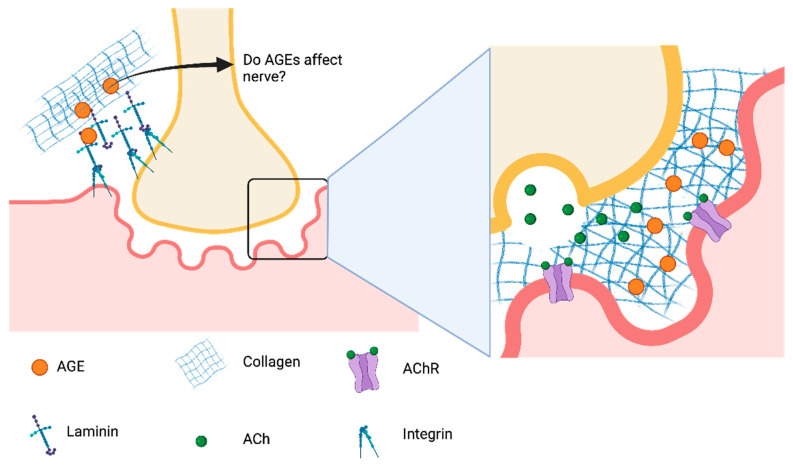
The role of AGEs at the neuromuscular junction are unclear and could have effects on synaptic vesicle release, acetylcholine diffusion to their receptors and an effect on signaling that may contribute to motor end-plate fragmentation and muscle aging.

While development of the NMJ is not completely understood, pre-patterning of acetylcholine receptors on the muscle membrane occurs before the motor nerve reaches the immature site [111]. Moreover, it is important to understand how the NMJ forms in order to address disruptions or degeneration of motor endplates due to disease or even AGEs (Figure 2). Interaction between motor nerves, leader Schwann cells, and skeletal muscle triggers the NMJ maturation and stabilization process. The pre-patterned acetylcholine receptors then enter a pruning phase and begin to consolidate to a centralized area on the muscle local to the motor neuron. Invaginations begin forming that increase the synaptic surface area while the acetylcholine receptors switch to mature subunits [112]. Additionally, during the maturation process, a carefully regulated extracellular matrix comprised heavily of collagen type IV and laminin develops in the region of the motor end plate, and is thought to contribute to NMJ development and maturation [113,114].

Laminins play major roles in motor neuron pathfinding and are known to be critical in synaptogenesis. Peripheral motor neurons traverse the body through their basal laminar tube comprised of laminin α2β1γ1 and α5β1γ1 [115]. Myelinating Schwann cells in the endoneurium cover the peripheral nerve and use the mechano-sensitive cues from the basal laminar tube for polarization and directionality [116]. Since mechanical queues play a critical role in Schwann cell pathfinding and subsequent regeneration, any disruptions to the connective tissue intramuscularly (e.g., AGE cross-linking) can drastically alter the peripheral nerve’s ability and speed of muscle innervation [117].

### 3.2. Endplate Fragmentation and Aging

Endplate fragmentation is a multi-stage disassociation of the NMJ typically seen during aging. This process is marked by a dispersal of clustered AChRs, AChR type switching, and increased turnover rates of the receptor [118,119]. As a way to compensate for fragmentation, increased enzyme levels of acetylcholinesterase and increased levels of acetylcholine (ACh) release from the synaptic terminal were detected [119]. Surprisingly, the receptor’s ability to bind to acetylcholine has not been shown to be inhibited with age, suggesting that ACh release into the synaptic cleft is a critical feature of endplate fragmentation and helps to explain increases in ACh release during fragmentation. Moreover, research from Carlson et al. indicated that fragmentation may play a part in fiber type switching due to age, a process referred to as homeostatic decompensation [44].

There is mounting evidence that the atrophy observed during aging is caused more by signaling disruptions than disuse. The disruption of cross-talk between the muscle and nerve can trigger downstream activation of atrogenes Murf-1 and Fbxo32 via TNF-α activation [120]. In more severe cases of atrophy like denervation, TNF-α synthesis increases, contributing to endplate breakdown. Similarly, circulating levels of TNF-α increase with age, and TNF-α is a major factor associated with endplate fragmentation and with activation of the RAGE pathway [121].

### 3.3. Insights from Diabetic Neuropathy, AGEs, and RAGE

**Figure 3:**
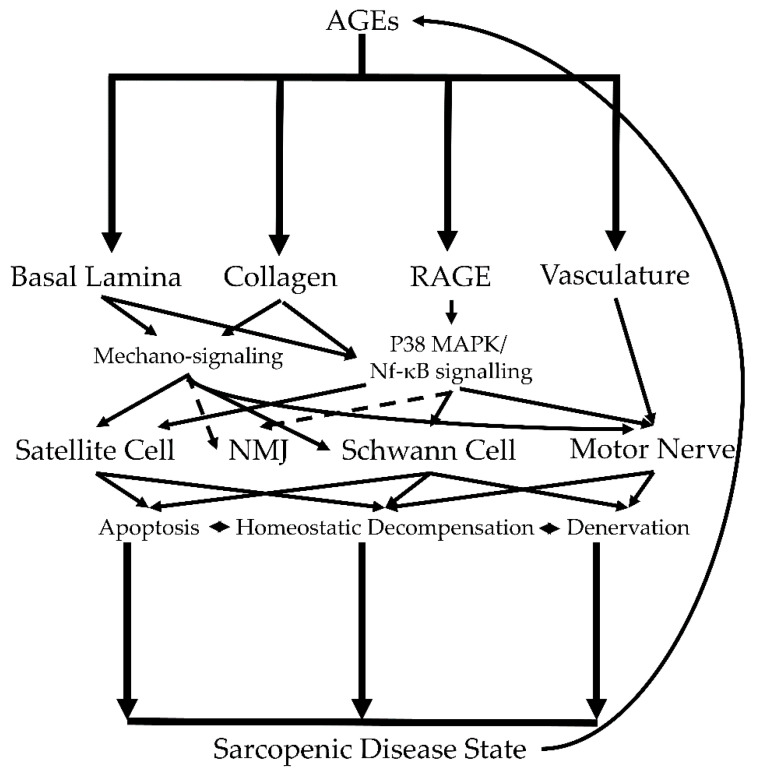
AGEs support a sarcopenic disease state in muscle by altering the basal lamina, collagen, RAGE signaling, and vasculature. These AGE-dependent changes accumulate and result in deleterious effects to satellite cells, NMJs, Schwann cells, and the motor nerve that culminate in sarcopenia. Known mechanisms are indicated by solid arrows while proposed mechanisms (future directions) are indicated with dashed arrows.

Diabetes may support an early onset aging phenotype [122,123]. Comparisons between diabetic muscle and aging muscle may be drawn, and aging is an independent factor that is predictive of diabetic neuropathy severity [122,123,124]. Similar to aging, diabetes also produces a pro-AGE environment in muscle and provides an alternative perspective for studying the impact of AGE-related changes. AGEs play a major role in diabetic neuropathy but its effects still need to be parsed [125,126]. Sensory nerves are highly affected by age-induced neuropathy, mirroring diabetic neuropathy [127]. Curiously, diabetic neuropathy causes the muscles of the distal limbs (foot to knee) of humans to undergo atrophy whereas the proximal limb (thigh muscles) preferentially undergo atrophy in sarcopenic individuals while the upper limbs in both conditions are relatively unaffected [128,129,130,131]. Further, AGE levels are correlated to sarcopenic muscle in the distal limb of older individuals with type 1 and type 2 diabetes [132,133]. However, it is unknown if the age-dependent atrophy of the lower limb is also driven by neuropathy or AGEs independent of diabetes. Like in diabetes, the number of motor units decrease in number but increase in size with age [134,135]. Intramuscular connective tissue increases in both aging and diabetes, and this is tied to AGE accumulation (Figure 3) [82,136]. There is untapped potential in using correlated knowledge on geriatric and diabetic motor nerve neuropathy to elucidate the role of AGEs in age-dependent changes to muscle-nerve interactions.

Hyperglycemic conditions in diabetes mellitus causes global AGE formation in the body, leading to diabetic neuropathy and a host of other complications [137]. Sensory neurons preferentially undergo dysfunctional Wallerian degeneration that leads to chronic denervation, and sensory nerve deficits are seen in 70% of diabetic patients [138]. In contrast, motor nerve deficits are seen in only 1–6% of patients and are confined to the distal limbs [139]. As such, much of the diabetic body of literature focuses on sensory neuropathy as this is the primary clinical concern, but the contributive impact of AGEs in diabetic neuropathogenesis needs further study. It has been posited that motor neurons are partially protected by the central nervous system blood–brain barrier from the glycolytic attack that sensory nerve cell bodies experience in the diabetic state [140,141]. Additionally, other compensatory mechanisms likely exist for motor nerves (e.g., single motor unit action potential enlargement) that sensory nerves lack [142].

Severe deficits in motor neurons are not seen until late in diabetic pathology where severe force deficits and motor neuron loss is observed [142]. However, there are minute, observable changes such as axonal sprouting, demyelination, and a decrease in both NMJ and motor unit number that occur in the beginning stages of diabetes [142,143]. AGEs destroy the microvasculature around motor nerves, slowing the conduction velocity [138,144,145,146]. However, there is little research into AGE’s direct effects on motor neuron diabetic neuropathy due to most of the literature concentrating on sensory neurons. In vitro study has been valuable in the effort to posit AGE-specific effects on sensory neurons. Rat sensory neurons grown on glycated collagen type IV, collagen type I, laminin, and fibronectin show a decrease in neurite outgrowth and dorsal root ganglion explants grown in high glucose conditions have decreased neurite outgrowth [147,148,149]. However, dorsal root ganglion explants from diabetic mice showed increased axonal sprouting, which better mirrors the in vivo reality where excessive axonal outgrowth interferes with successful re-innervation [150,151,152,153,154]. Moreover, AGEs are known to accumulate in the peripheral endonerium in diabetic mice [148]. Whether these results translate to motor neurons is not known. Additionally, it is known that AGEs are highly cytotoxic to Schwann cells via the P38 MAPK and NF-κB pathways [149,155,156,157,158,159]. Further, it is clear that the motor nerve neuropathy can be driven by AGE cross-linked ECM components.

## 4. Future Directions

The research covered in this review reveals multiple gaps in our current knowledge regarding AGEs, atrophy, and denervation. Measuring AGEs is particularly difficult since most of the AGE cross-links reside in the irreducible matrix. Methods to measure AGEs need to be standardized, and incorporating ECM normalization factors such as hydroxyproline in homogenization schemas could take into account the insoluble AGEs [82]. There is much work to be done in understanding the integrative role of both mechanical stiffening and RAGE signaling due to AGE cross-links on skeletal muscle biology as most of the literature focuses on the effects of soluble AGEs alone. Additionally, the role of AGE cross-links on satellite cell aging and the aging of the satellite cell niche needs to be elucidated, with particular attention to basal lamina remodeling and stiffening. Further efforts to distinguish the roles of the normal LOX-dependent cross-links in old age would serve to better understand the role of pathological age-dependent AGE cross-links. While it is known that AGEs play a role in peripheral nerve neuropathy, it is not well characterized if AGEs promote a denervated phenotype in aged muscle, or how much they contribute to the atrophic environment in sarcopenic muscle. Studies elucidating the role of AGE-cross-linked collagen on motor nerve health need to be developed.

Muscle and nerve signaling and their relation to the ECM has been particularly difficult to study due to the plastic sensitivity of innervation and inability to study it ex vivo. To date, the current standard for studying muscle-nerve interactions and endplate remodeling is in animal models. There is currently no in vitro model that accurately re-creates muscle innervation. Development of such a model would greatly improve reproducibility while reducing the noise, expense, and animal sacrifice currently required. Further, while there has been increased interest in understanding the specialized endplate ECM, few papers have been published on the non-myelinating Schwann cells that cover each endplate and are known to protectively regulate the intra-synaptic ECM. It is unknown if AGEs cross-link within the synaptic cleft or how cross-linking this specialized ECM effects acetylcholine transmission. Hence, this area of research is relatively unexplored, leaving a possible future topic in ECM modifications and muscle to nerve signaling during aging and other diseases that increase AGE cross-links. In addition, whether modifications to laminin change neural pathfinding and synapse formation is still unclear and interrogating these types of questions might lead to new discoveries in endplate development, motor nerve pathfinding, and endplate stability in the neuromuscular junction.

## 5. Conclusions

Skeletal muscle aging is a process influenced by deterministic and stochastic processes, and is characterized by muscle wasting, fast-to-slow muscle fiber transition, and an increase in the skeletal muscle collagens. AGE collagen cross-linking clearly plays a role in sarcopenia; however, much study is warranted in further elucidating the impact of AGEs in skeletal muscle. Furthermore, age-related changes to motor nerves drive much of sarcopenia, and may involve AGE cross-linking.
